# Changes in Morphological and Physicochemical Properties of Waxy and Non-waxy Proso Millets during Cooking Process

**DOI:** 10.3390/foods8110583

**Published:** 2019-11-17

**Authors:** Qinghua Yang, Long Liu, Weili Zhang, Jing Li, Xiaoli Gao, Baili Feng

**Affiliations:** State Key Laboratory of Crop Stress Biology for Arid Areas, College of Agronomy, Northwest A & F University, Yangling 712000, China; 2016060037@nwsuaf.edu.cn (Q.Y.); 17395074649@163.com (L.L.); zhang1415203881@163.com (W.Z.); lijing1993@nwafu.edu.cn (J.L.); gao2123@nwsuaf.edu.cn (X.G.)

**Keywords:** proso millet, amylose, physicochemical properties, cooking process

## Abstract

Proso millet, a grain which is principally consumed in cooked form, is favored by consumers because of its rich nutritional value. However, the changes in morphological and physicochemical properties of proso millet grains occurring during the cooking process have rarely been reported. In this study, we investigated the changes in morphological and physicochemical properties of cooked waxy and non-waxy proso millets. During the cooking process, starch granules in the grains were gradually gelatinized starting from the outer region to the inner region and were gelatinized earlier in waxy proso millet than in non-waxy proso millet. Many filamentous network structures were observed in the cross sections of cooked waxy proso millet. As the cooking time increased, the long- and short-range, ordered structures of proso millets were gradually disrupted, and the ordered structures were fully disrupted by 20 min of cooking. In both waxy and non-waxy proso millets, thermal and pasting properties significantly changed with an increase in the cooking time. This study provides useful information for the processing of proso millet in the food industry.

## 1. Introduction

The grains of proso millet (*Panicum miliaceum* L.) have higher levels of proteins, several minerals, vitamins, and antioxidants than most other cereals [1]. In addition, it is gluten-free and suitable for individuals with gluten allergies [2]. For nutritional balance, people often add “Huang Mi” (shelled proso millet grains) to rice when cooking [3]. Some studies of proso millet have been reported: some of these have focused on germplasm resources [4], drought-resistant mechanisms [5], highly efficient cultivations [6], and functional ingredients [7]. However, the cooking quality of proso millet, a key property of concern to consumers, plays an important role in the production, processing, and application of this grain, and relevant studies regarding this are scarce.

Cereals such as rice and millet are principally consumed as cooked grains [3,8]. During cooking, starch granules in cereal grains gelatinize as they absorb water [8]. The effect of cooking on cereal grains [9,10], including the characteristics of cooked grains, such as texture and digestive properties, is a key factor affecting consumer purchasing decisions. Previous studies indicate that the cooking of rice is affected by many factors, including temperature and time [11], water content [12], high-pressure steam [8], and amylose content [11]. Understanding the changes occurring in cereal grains during the cooking process is the basis for investigating the effects of cooking conditions on cooking quality and further promoting the consumption of cereals. Pan et al. [9] studied morphological changes in kernels and the starch properties of high-amylose rice lines, during cooking in boiling water. He et al. [11] investigated the impact of cooking temperature and time on the properties of rice. Xu et al. [8] reported the effects of high-pressure steam on the eating quality of cooked rice. However, compared with those of rice, the morphological and physicochemical changes in proso millet grains during the cooking process have rarely been reported.

Starch is the most important component of proso millet grains. Based on the content of amylose, proso millets can be divided into waxy and non-waxy varieties [13]. The grain quality properties of waxy and non-waxy proso millets exhibit significant differences [3]. Starch granules in proso millet with different amylose contents also have different physicochemical properties [14,15]. Studying the changes occurring in proso millet during the cooking process can help further explain differences in grain qualities.

We investigated the chemical composition, morphological properties, crystalline structure, ordered structure, thermal properties, and pasting properties of waxy and non-waxy proso millets during the cooking process. The objective was to reveal differences in the morphological and physicochemical properties of waxy and non-waxy proso millet grains occurring during the cooking process.

## 2. Materials and Methods

### 2.1. Materials

Waxy (Yushu 1) and non-waxy (Chimi 2) proso millet varieties were grown in the Experimental Fields of the Northwest A&F University (Yulin, Shaanxi, China) in 2018. Mature seeds were shelled using a huller (SY88-TH, SsangYong machinery factory, Seoul Korea) and stored at 4 °C.

### 2.2. Cooking Conditions and Sample Preparation

Fifty proso millet grains were randomly selected and rinsed with 10 mL of water. Subsequently, the grains were placed in a 25 mL beaker containing 20 mL of water for 10 min. A 1300-W electric cooker containing 3 L of water was brought to a boil, and the beaker was placed on a shelf in the cooker and steamed. The beaker was then transferred into an ice bath at 5 min interval and was allowed to cool for 2 min to prevent further cooking of proso millet grains. The total cooking time was 30 min. The waxy proso millet grains that were cooked for 5, 10, 15, 20, 25, and 30 min were represented by W1, W2, W3, W4, W5, and W6, respectively; the non-waxy proso millet grains were cooked for 5, 10, 15, 20, 25, and 30 min were represented by N1, N2, N3, N4, N5, and N6, respectively. Uncooked waxy (W0) and non-waxy (N0) proso millet grains were used as blank control. Some cooled samples were used to determine the characteristics of cooked proso millet grains. Other proso millet grains in beakers were washed with 20 mL of water and then freeze-dried (LGJ-10, SongYuanXingHua Ltd., Beijing, China), pulverized into flour, and filtered through a 100-mesh sieve.

### 2.3. Determination of Cooking Characteristics

The cooled, cooked proso millet grains were pressed between two clean glass plates to measure the degree of cooking. Excess, residual cooking water was transferred to a dry aluminum box. The cooked grains were washed once with distilled water, and the water obtained after rinsing was transferred to the dry aluminum box. Finally, the collected water was dried to constant weight.

Water present on the surface of the grains was removed using a filter paper, and the volume and weight of grains were determined using the volume displacement method by draining water and the weighing method, respectively. The expansion ratio and water absorption ratio were defined as the percentage increase in grain volume and weight after cooking relative to the volume and weight of uncooked grains, respectively.

Next, 5.0 mL of 0.5 mol/L HCl and 1.0 mL of 0.2% iodine reagent were added to 1.0 mL of cooking water and then additional water was added to make up the volume to 100 mL. Finally, the iodine blue value was datermined using a spectrophotometer at a wavelength of 660.0 nm.

### 2.4. Chemical Compositions

The fat and protein content of the grains were determined using Soxhlet extraction (Solvent: petroleum ether (boiling point, 60 °C–90 °C)) and the Kjeldahl method (conversion coefficient of nitrogen, 6.25) [14], respectively. Amylose and total starch contents were determined in accordance with the protocols of an amylose test kit and a total starch test kit (Megazyme Co., Ltd., Bray, Ireland), respectively.

### 2.5. Morphological Properties of Proso Millet Grains

The morphological characteristics of waxy and non-waxy proso millet grains before and after cooking were observed using scanning electron microscopy (SEM; S-4800, Hitachi Limited, Tokyo, Japan). After cooking for 5, 10, 15, 20, 25, and 30 min, five grains of each variety at each time point were selected for observation, and five uncooked grains were used as controls. The cross sections of the grains were obtained by fracturing them along their embryo using a single-sided blade. The samples were soaked in 4% glutaraldehyde and placed in a refrigerator at 4 °C overnight, rinsed four times with 0.1-M phosphate buffer, dehydrated with by 30%, 50%, 70%, 80%, 90%, and 100% (*v/v*) ethyl alcohol, and then transferred to 5 mL isoamyl acetate (24 h). Subsequently, the samples were dried using the critical-point drying method (K850, Quorum Technologies Ltd., East Sussex, England), and observed at a magnification of 2000× [16].

### 2.6. Morphological Characteristics of Proso Millet Flour

The morphology of both proso millet flours was observed using SEM according to a method reported by Yang et al. [17].

### 2.7. Crystalline Structure

The crystalline structure of proso millet flour was studied using D8 X-ray diffraction (XRD) (Bruker, Falkenried, Germany) at a target voltage of 40 kW, a target current of 100 mA, a scan range of 5–50° (2θ), and a scanning speed of 10.0° /min.

### 2.8. Short-Range Ordered Structure

The short-range ordered structure of the external region of starch granules in proso millet flour was analyzed using a Fourier transform infrared (FTIR) spectrometer (Nicolet iS50, Thermo Fisher Scientific, Waltham, MA, USA) [18]. Thirty-two scans were obtained at a resolution of 4 cm^−1^. The ratio of absorbance 1045/1022^−1^ and 1022/995 cm^−1^ were calculated for analyzing the short-range ordered structure of starches in flour.

### 2.9. Thermal Properties

According to the method reported by Yang et al. [17], the thermal properties of proso millet flour were analyzed using a differential scanning calorimeter (Q 2000, TA Instruments Inc, Newcastle, DE, USA).

### 2.10. Pasting Properties

The pasting properties of proso millet flour were measured using a rapid visco analyzer (RVA 4500, Perten, Stockholm, Sweden) according to the method reported by Yang et al. [17] with following modifications: 3 g of proso millet flour was used with 20 mL of water.

### 2.11. Statistical Analysis

Sample measurements were performed three times and all data were represented as means ± standard deviations. Data were analyzed using SPSS 16.0 (SPSS Inc., Chicago, IL, USA), and Duncan’s multiple range tests (*p* < 0.05) were performed to analyze differences between measurements.

## 3. Results and Discussion

### 3.1. Proso Millet Grain Morphology during the Cooking Process

**Figure 1:**
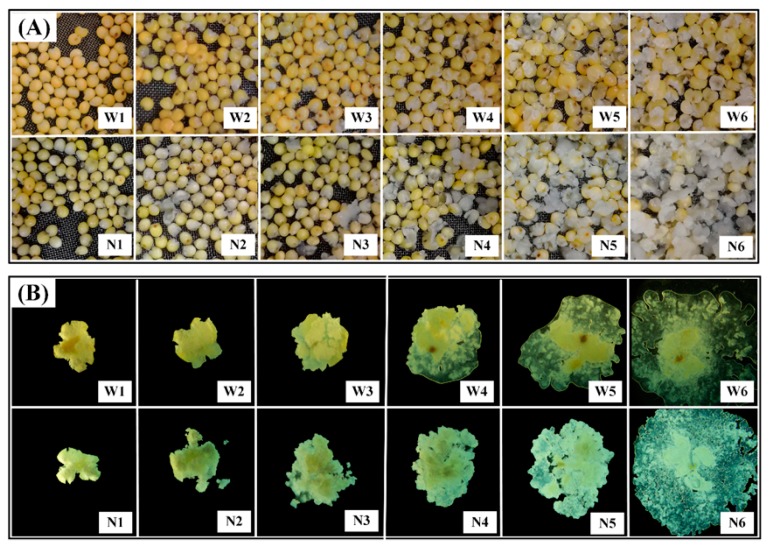
Morphology pictures of proso millet grains at different cooking times (**A**), and proso millet grains at different cooking times pressed between two glass slides during (**B**). W0–W6: uncooked, and cooked for 5, 10, 15, 20, 25, and 30 min, respectively; N0–N6: Uncooked and cooked for 5, 10, 15, 20, 25, and 30 min, respectively.

The morphological properties of waxy and non-waxy millets changed with varying degrees during the cooking process (Figure 1A). The deformation in non-waxy proso millet grains was more severe than that in waxy proso millet grains. Pan et al. [9] reported that high-amylose-containing grains demonstrated less deformation, which was different from our results. In our study, complete gelatinization of non-waxy proso millet grains required more time than waxy proso millet grains; however, the degree of deformation was greater in the former. This difference may be attributed to crops, genotypes, or the physical appearance of the grains.

The degree of gelatinization after being pressed between two glass slides is shown in Figure 1B. Waxy and non-waxy proso millet grains showed initial gelatinization after 10 and 15 min of cooking and complete gelatinization after 20 and 25 min of cooking, respectively, indicating that non-waxy proso millet grains were more resistant to cooking than waxy proso millet grains. A study on rice [9] has indicated that thorough cooking of indica rice requires 30 min while that of high-amylose rice requires >60 min, which is consistent with our results of proso millet grains. Compared with rice, proso millet grains took less time to undergo complete gelatinization, which may be due to the relatively smaller size of proso millet grains. In addition, high-amylose varieties require more time to completely gelatinize, likely because amylose is more difficult to gelatinize than amylopectin [19].

**Figure 2:**
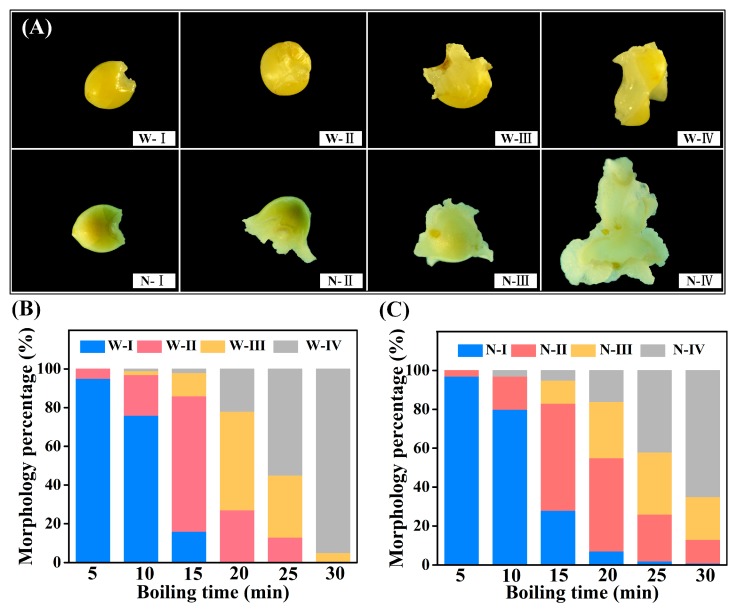
Representative morphologies of cooked proso millet grains (**A**) and the percentage of the morphological types of waxy (**B**) and non-waxy (**C**) proso millet grains. W-I-W-IV: Four morphology types of waxy proso millet grain; N-I-N-IV: Four morphology types of non-waxy proso millet grain.

Cooked waxy and non-waxy proso millet grains were both classified into four morphological types: undeformed, slightly deformed, greatly deformed, and completely deformed (W-I-W-IV; and N-I-N-IV) (Figure 2A). The percentages of each morphological type of proso millet grains at each boiling time were summarized in Figure 2B,C. As the cooking time increased, the percentage of severely deformed grains gradually increased. At the last time point (30 min), 95% and 5% of waxy proso millet grains were completely and greatly deformed, respectively. However, in non-waxy proso millet grains, the percentages of completely, greatly, and slightly deformed grains after cooking for 30 min were 65%, 22%, and 12%, respectively, which indicated that different morphology grains exist simultaneously in this period. Therefore, ensure non-waxy proso millet grains were gelatinized together is one of the problem in the processing of non-waxy proso millet.

### 3.2. Characteristics of Cooked Proso Millet Grains

**Figure 3:**
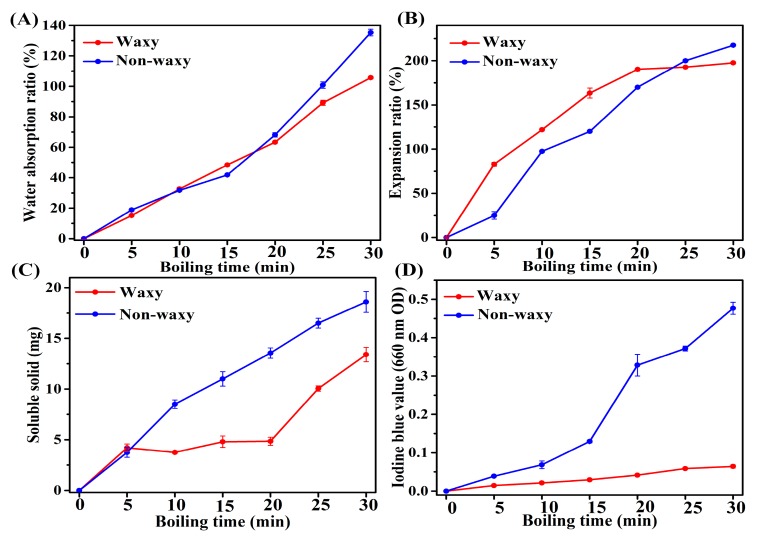
Cooking characteristics of proso millet. (**A**) Water absorption ratio, (B) expansion ratio, (**C**) soluble solid, and (**D**) iodine blue value, respectively.

The characteristics of cooked proso millet grains are presented in Figure 3. As the cooking time increased, the water absorption ratio, expansion ratio, soluble solid content, and iodine blue value of both waxy and non-waxy proso millet grains gradually increased. Water absorption and gelatinization of proso millet grains led to increases in volume and weight, which in turn led to the leaching of materials from grains [11]. Finally, the water absorption ratio, expansion ratio, and soluble solids content of non-waxy proso millet grains were higher than those of waxy proso millet grains, which indicated that the porridge made from non-waxy proso millet will be thicker. In contrast to the present study, some studies have reported that rice with low amylose showed greater structural changes after cooking [20], with amylose hindering water intake during cooking [21,22]. This may be due to the fact that non-waxy proso millet grains were more seriously damaged than waxy proso millet grains. In addition to amylose content, amylopectin chain length, fat content, protein content, and the thickness of the outer layer of the grain all affect the characteristics of cooked grains [23,24]. These factors may also be responsible for the differences in the characteristics of cooked rice and proso millet grains.

### 3.3. Chemical Compositions of Proso Millet Flour

The fat, protein, total starch, and amylose contents of uncooked waxy proso millet flours were 3.91%, 16.85%, 64.41%, and 4.27% and those of uncooked non-waxy proso millet flour were 3.58%, 14.74%, 66.48%, and 28.85%, respectively (Table 1). As the cooking time increased, the fat content of both the flours decreased and the protein content increased.

### 3.4. SEM Images of Proso Millet Grains and Flours

**Figure 4:**
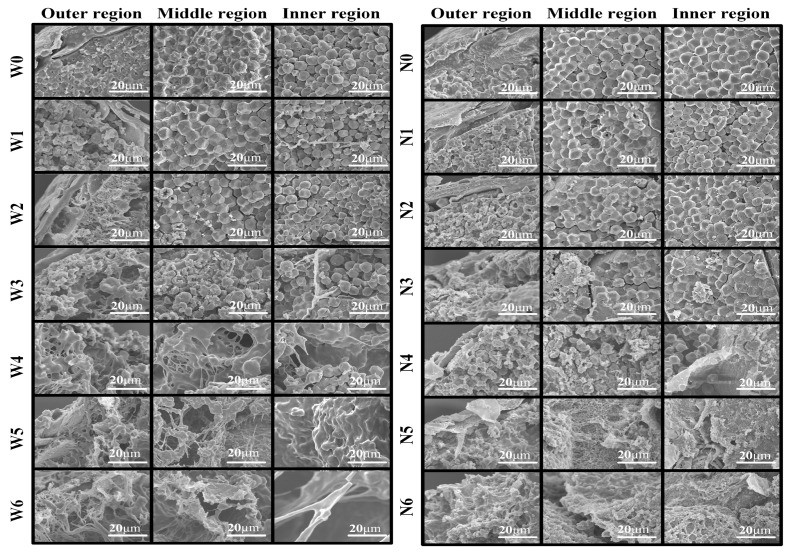
Micrographs of different regions in uncooked and cooked waxy and non-waxy proso millet grains. W0–W6: uncooked, and cooked for 5, 10, 15, 20, 25, and 30 min, respectively; N0–N6: Uncooked and cooked for 5, 10, 15, 20, 25, and 30 min, respectively.

Starch is the most important component of proso millet grains. During the cooking process, gelatinization of starch granules is achieved by three processes: swelling, morphological change, and adhesion [9]. Morphological changes in the outer, middle, and inner regions of waxy and non-waxy proso millet grains are shown in Figure 4. The surface of uncooked waxy and non-waxy proso millet grains was found to be closely connected to starch; moreover, starch granules in the middle and inner regions were neatly arranged, forming a regular polygonal structure. After cooking for 5 min, starch granules in the outer region of waxy proso millet grains appeared swollen or damaged. These granules were found to be completely damaged, sticking together to form a honeycomb structure in 15 min. Fifteen minutes later, starch granules in the outer, middle, and inner regions of waxy proso millet grains had formed branched structures, appeared swollen or damaged, and were slightly swollen, respectively. Behind 15 min, starch granules in the outer, middle, and inner regions of waxy proso millet grains all formed branched structures. However, some broken starch granules were also observed. As the cooking time increased, the branches elongated, especially those in the inner region, which may be due to the cracking of proso millet grains [25].

When cooking for 5 and 10 min, starch granules in the outer region of non-waxy proso millet grains were slightly swollen and severely damaged, respectively. Behind 15 min, starch granules in the outer, middle, and inner regions of non-waxy proso millet grains were completely damaged, began to swell or damage, and were slightly swelled, respectively. After cooking for 20 min, starch granules in the middle and inner regions of waxy proso millet grains were severely damaged, while some intact starch granules were still observed in the inner region. Finally, starch granules in the outer, middle, and inner regions all showed honeycomb structures in 25 min and 30 min. The morphological differences between waxy and non-waxy proso millet grains may be attributed to the differences in amylose content [26]. In addition, starch granules in non-waxy proso millet grains were damaged later than those in waxy proso millet grains, indicating that starch granules in the former were more resistant to gelatinization [14].

**Figure 5:**
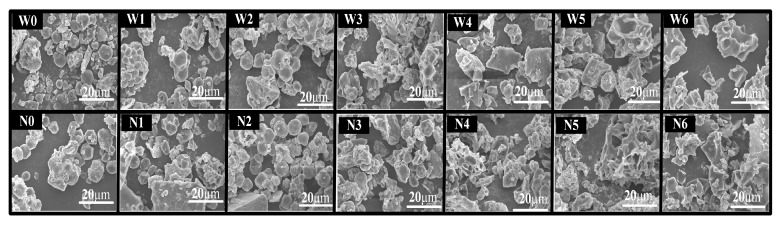
Micrographs of flours in uncooked and cooked proso millet grains. W0–W6: Uncooked and cooked (5, 10, 15, 20, 25, and 30 min) waxy proso millet grains, respectively; N0–N6: Uncooked and cooked (5, 10, 15, 20, 25, and 30 min) non-waxy proso millet grains, respectively.

The morphology of uncooked and cooked proso millet flours was determined and compared (Figure 5). After cooking for 10 min, starch granules in waxy and non-waxy proso millet flours began to swell. After cooking for 15 min, starch granules in waxy proso millet flour were more seriously damaged than those in non-waxy proso millet flours. After cooking for 20 min, starch granules disappeared from waxy proso millet flours, while some damaged starch granules were still evident in non-waxy proso millet flours. After a further increase in cooking time to 25 and 30 min, irregular polyhedral structures with holes were observed in both waxy and non-waxy proso millet flours.

### 3.5. Crystalline Structure

**Figure 6:**
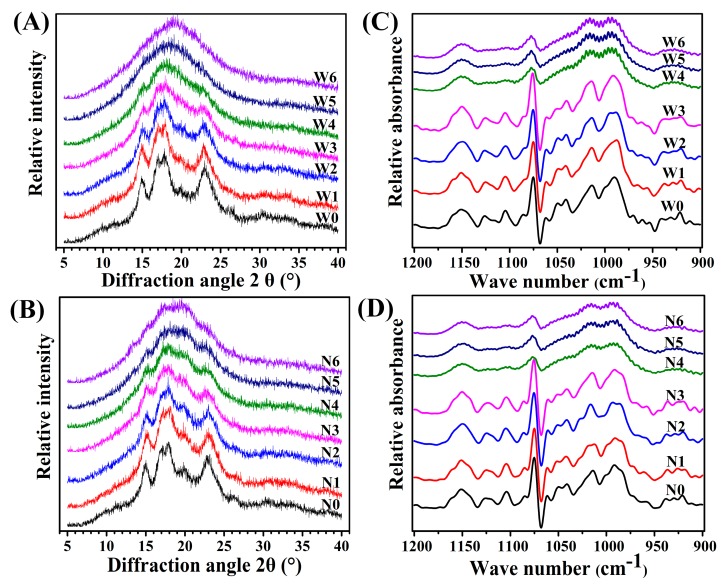
X-ray diffraction patterns (XRD) (**A, B**) and ordered structure (FTIR) (**C, D**) of uncooked and cooked proso millet flours. W0–W6: Uncooked and cooked (5, 10, 15, 20, 25, and 30 min) waxy proso millet flour, respectively; N0–N6: Uncooked and cooked (5, 10, 15, 20, 25, and 30 min) non-waxy proso millet, respectively.

The XRD profiles of waxy and non-waxy proso millet flours before and after cooking are shown in Figure 6A,B. Uncooked proso millet flours exhibited two single peaks at 15° and 23° 2θ and a continuous double peak at approximately 17° and 18° 2θ, with raw proso millet flour displaying typical A-type diffraction patterns. The peak intensity of both proso millet flours gradually decreased with an increase in the cooking time. The crystalline structure of the starch granules of waxy proso millet flour was completely destroyed earlier than that of the starch granules of non-waxy proso millet flour (cooking time, 20 and 25 min, respectively). This was consistent with the results observed using SEM. In addition, the XRD patterns of flours fluctuated and were not particularly smooth compared with those of starches in proso millet grains [14], possibly because the flours contained more components. Uncooked proso millet flours showed a weak peak at approximately 20° 2θ, which some studies [27,28] have reported to be the diffraction peak of an amylose–lipid complex. Furthermore, the peak at 20° 2θ in non-waxy proso millet flour was more remarkable than that in waxy proso millet flour, which can be attributed to the higher amylose content of non-waxy proso millet, facilitating the creation of amylose–lipid complexes. As the cooking time increased, this peak (20° 2θ) gradually became larger and eventually formed a characteristic V-type polymorph peak. This demonstrated that cooking promoted the formation of amylose–lipid complexes, which is consistent with other studies [9,27,29,30].

### 3.6. Ordered Structure

The FTIR spectra, 1045/1022 cm^−1^ ratio, and 1022/995 cm^−1^ ratio of the flours are shown in Figure 6C,D and Table 1. The FTIR spectra of the flours were sensitive to short-range ordered structure (double-helical order); moreover, XRD can detect long-range ordered structure (crystallinity) [31]. Reportedly, 1045/1022 cm^−1^ and 1022/995 cm^−1^ ratios are used as a measure of the ordered degree and the proportion of amorphous-to-ordered carbohydrate structure in starch granules, respectively [32]. In our study, 1045/1022 cm^−1^ and 1022/995 cm^−1^ ratios ranged from 0.345 to 0.420 and from 0.735 to 0.889, respectively (Table 1). In sweet potato starches [33] and fruit kernel starches [34] 1045/1022 cm^−1^ ratio has been reported to be 0.631–0.676 and 0.576–0.654, respectively, and these differences may be due to crop types. After cooking, 1045/1022 cm^−1^ ratio decreased and 1022/995 cm^−1^ ratio increased, indicating a gradual damage to the ordered structure. After cooking for 20 min, absorption peaks in waxy and non-waxy proso millet flours both disappeared (Figure 6C,D).

### 3.7. Thermal Properties of Proso Millet Flours

**Figure 7:**
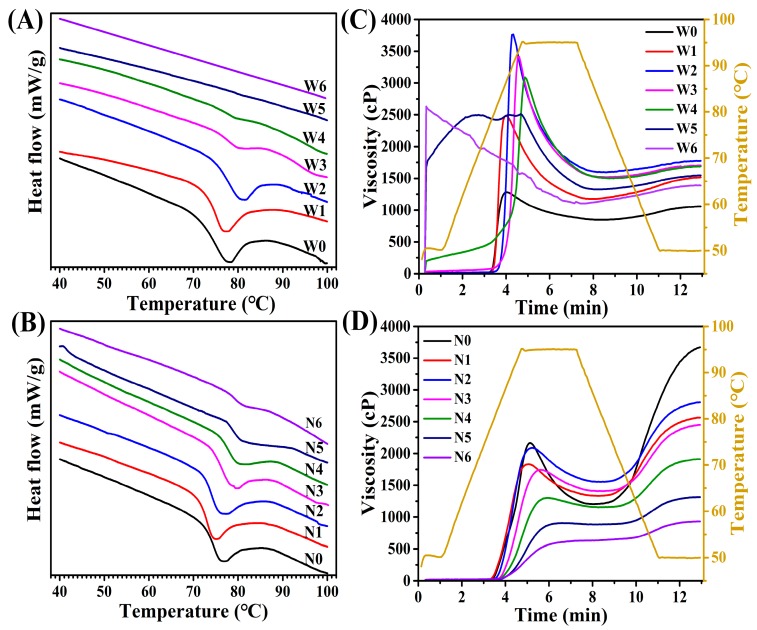
Thermal property (DSC) curves (**A, B**) and pasting property (RVA) patterns (**C, D**) of uncooked and cooked proso millet flours. W0–W6: Uncooked and cooked (5, 10, 15, 20, 25, and 30 min) of waxy proso millet flour, respectively; N0–N6: Uncooked and cooked (5, 10, 15, 20, 25, and 30 min) non-waxy proso millet flour, respectively.

The thermal properties of uncooked and cooked proso millet flours are summarized in Table 2 and Figure 7A,B. The onset temperature (To) of uncooked waxy proso millet flour was lower than that of uncooked non-waxy proso millet flour, whereas the peak temperature (Tp), conclusion temperature (Tc), and gelatinization enthalpy (ΔH) of cooked waxy proso millet flour were higher than those of uncooked non-waxy proso millet flour. A higher gelatinization temperature represents a more stable crystal structure, and a higher ΔH indicates the requirement of more energy to melt starch granules [35]. Some researchers have reported that the starch granules of waxy proso millet have more amylopectin to exhibit greater crystallinity than those of non-waxy proso millet; thus, the gelatinization of starches in waxy proso millet flour requires higher temperatures and more energy than that in non-waxy proso millet flour [14]. Except To, the results of Tp, Tc, and ΔH were consistent with previous reports. This was may be because the present study concentrated on flours rather than starches, and the additional components in flours may affect the results.

The gelatinization temperature and ΔH of proso millet flours demonstrated a reasonably wide gap with a change in the cooking time. As the cooking time increased, the gelatinization temperatures of proso millet flours significantly increased, while the ΔH of proso millet flours significantly decreased, which is in agreement with previous studies [11,27]. However, unstable starch crystallites melted first, with the remaining stable crystallites requiring higher temperatures to melt [36]. Moreover, when the cooking time increased, the melting of starch crystallites and the formation of amylose–lipid complexes resulted in a decrease in enthalpy [37]. The gelatinization temperature and ΔH could not be detected when the cooking time was 20 and 25 min for waxy and non-waxy proso millet flours, respectively; this indicates that the complete gelatinization of waxy proso millet flour occurred earlier than that of non-waxy proso millet flour.

### 3.8. Pasting Properties of Proso Millet Flour

The peak viscosity (PV) and breakdown viscosity (BD) of flour have important impact on food processing, appearance, texture, and taste [14]. The pasting properties of proso millet flours are presented in Table 3 and Figure 7C,D. The peak viscosity (PV), trough viscosity (TV), BD, FV, setback viscosity (SB), peak time (PT), and pasting temperature (PTM) of uncooked non-waxy proso millet flour were significantly higher than those of uncooked waxy proso millet flour. In the industry, the processing of non-waxy proso millet requires higher temperature. Therefore, the non-waxy proso millet is suitable for making high-temperature processed food and the waxy proso millet is suitable for making frozen fast food. However, some studies [14] have reported that starch granules in waxy proso millet flour had higher PT, TV, BD, and FV, which may be due to the fact that components, such as fat and protein, in the flour may affect gelatinization properties. The PV, TV, FV, and SB of proso millet flours first increased and then decreased with the cooking time. This is because the swelling of cooked flour resulted in increased PV. With an increase in the cooking time, the swelling gradually saturated. Further cooking led to damaged and swollen starch granules and the formation of amylose–lipid complexes, resulting in a decreased PV [11,38]. These values start to decrease 5 min ahead for waxy proso millet flours. Interestingly, after cooking for 30 min, the PV, TV, FV, and SB of waxy proso millet flour significantly increased and PT and PTM greatly reduced compared with those of uncooked flour. For the non-waxy proso millet flour, PV, TV, FV, and SB significantly reduced, while the PT and PTM greatly increased. This intriguing opposite trend may be due to the difference in amylose/amylopectin [39] or the ability of the starch granules in proso millet to bind to water [12].

## 4. Conclusions

Cooking changed the morphological and physicochemical properties of both waxy and non-waxy proso millets. The water absorption ratio, expansion ratio, soluble solid, and iodine blue value of cooked proso millets increased with the cooking time, and the water absorption ratio, soluble solid, and iodine blue value were more pronounced in non-waxy proso millets than in waxy proso millets. Starch granules in proso millet grains were gradually gelatinized from the outer region to the inner region with cooking time, and the starch granules in waxy proso millet were gelatinized earlier than those in non-waxy proso millet. Compared with those of cooked non-waxy proso millet, many filamentous network structures were observed in the cross sections of cooked waxy proso millet. As the cooking time increased, the long-range and short-range ordered structures of proso millets were gradually disrupted. The ordered structures were completely disrupted after cooking for 20 min. With an increase in the cooking time, the gelatinization temperature and ΔH of proso millet flours significantly increased and decreased, respectively. The gelatinization temperature and ΔH could not be observed after a cooking time of 20 min for waxy proso millet and 25 min for non-waxy proso millet. As the cooking time increased, the pasting properties of both the proso millet flours significantly changed and the variation trends for waxy and non-waxy proso millet differed.

## Figures and Tables

**Table 1:** Chemical compositions and IR ratio of uncooked and cooked flours.

Samples	Chemical Compositions Content (%)				IR Ratio	
	Fat	Protein	Amylose	Total Starch	1045/1022 (cm^−1^)	1022/995 (cm^−1^)
W0	3.91 ± 0.13 ^a^	16.85 ± 0.09 ^cd^	4.27 ± 0.11 ^f^	64.41 ± 1.46 ^ab^	0.420 ± 0.010 ^a^	0.735 ± 0.003 ^f^
W1	3.82 ± 0.13 ^ab^	16.72 ± 0.05 ^d^	4.34 ± 0.21 ^f^	64.00 ± 1.07 ^b^	0.404 ± 0.007 ^a^	0.800 ± 0.009 ^d^
W2	3.65 ± 0.10 ^ab^	16.77 ± 0.10 ^cd^	4.51 ± 0.16 ^f^	64.23 ± 1.51 ^ab^	0.381 ± 0.002 ^b^	0.818 ± 0.011 ^c^
W3	3.23 ± 0.18 ^cd^	16.70 ± 0.06 ^d^	4.42 ± 0.19 ^f^	64.43 ± 1.14 ^ab^	0.378 ± 0.008 ^b^	0.844 ± 0.009 ^b^
W4	3.14 ± 0.20 ^d^	16.95 ± 0.18 ^c^	4.26 ± 0.17 ^f^	63.83 ± 1.20 ^b^	n.d.	n.d.
W5	3.18 ± 0.20 ^d^	17.21 ± 0.06 ^b^	3.97 ± 0.09 ^f^	63.63 ± 1.43 ^b^	n.d.	n.d.
W6	2.92 ± 0.13 ^de^	17.67 ± 0.01 ^a^	3.90 ± 0.13 ^f^	63.13 ± 1.73 ^b^	n.d.	n.d.
N0	3.58 ± 0.10 ^b^	14.74 ± 0.10 ^h^	28.85 ± 1.27 ^cd^	66.48 ± 1.00 ^ab^	0.387 ± 0.005 ^b^	0.776 ± 0.006 ^e^
N1	3.54 ± 0.09 ^bc^	14.67 ± 0.13 ^h^	26.84 ± 0.59 ^d^	66.29 ± 1.24 ^ab^	0412 ± 0.007 ^a^	0.783 ± 0.008 ^de^
N2	3.25 ± 0.16 ^cd^	14.86 ± 0.06g ^h^	32.74 ± 1.52 ^b^	66.53 ± 1.88 ^ab^	0.372 ± 0.004 ^b^	0.824 ± 0.008 ^c^
N3	2.93 ± 0.12 ^de^	14.86 ± 0.13g ^h^	37.93 ± 0.95 ^a^	67.89 ± 1.82 ^a^	0.345 ± 0.006 ^c^	0.889 ± 0.003 ^a^
N4	2.76 ± 0.12 ^ef^	15.04 ± 0.06 ^g^	24.38 ± 1.34 ^e^	65.49 ± 2.16 ^ab^	n.d.	n.d.
N5	2.51 ± 0.14 ^fg^	15.39 ± 0.04 ^f^	29.22 ± 1.64 ^c^	66.19 ± 1.78 ^ab^	n.d.	n.d.
N6	2.36 ± 0.10 ^g^	15.61 ± 0.07 ^e^	32.25 ± 1.76 ^b^	64.81 ± 1.18 ^ab^	n.d.	n.d.

Data represent means ± standard deviations. For each column, values not displaying the same letter are significantly different (*p* < 0.05). W0–W6: uncooked, and cooked for 5, 10, 15, 20, 25, and 30 min, respectively; N0–N6: Uncooked and cooked for 5, 10, 15, 20, 25, and 30 min, respectively. IR: Infrared radiation; n.d.; Not detected.

**Table 2:** Thermal properties of uncooked and cooked flours.

Samples	To (°C)	Tp (°C)	Tc (°C)	ΔH (J/g)	Tc-To (°C)
W0	71.1 ± 0.1 ^d^	77.1 ± 0.1 ^d^	83.1 ± 0.8 ^b^	7.5 ± 0.7 ^a^	12.0 ± 0.1 ^a^
W1	72.8 ± 0.2 ^c^	76.6 ± 0.2 ^e^	82.6 ± 0.0 ^b^	6.3 ± 0.3 ^b^	9.8 ± 0.2 ^b^
W2	74.9 ± 0.1 ^b^	80.1 ± 0.1 ^a^	85.5 ± 0.4 ^a^	5.3 ± 0.1 ^c^	10.6 ± 0.3 ^b^
W3	75.6 ± 0.0 ^a^	78.9 ± 0.2 ^b^	84.3 ± 0.1 ^a^	0.6 ± 0.0 ^h^	8.7 ± 0.0 ^c^
W4	n.d.	n.d.	n.d.	n.d.	n.d.
W5	n.d.	n.d.	n.d.	n.d.	n.d.
W6	n.d.	n.d.	n.d.	n.d.	n.d.
N0	72.2 ± 0.3 ^c^	75.6 ± 0.2 ^f^	80.9 ± 0.7 ^c^	4.3 ± 0.1 ^d^	8.6 ± 1.0 ^c^
N1	70.8 ± 0.8 ^d^	74.4 ± 0.3 ^g^	78.6 ± 0.8 ^d^	3.8 ± 0.2 ^e^	7.8 ± 0.1 ^cd^
N2	72.6 ± 0.4 ^c^	75.4 ± 0.1 ^f^	80.8 ± 0.2 ^c^	4.0 ± 0.1 ^e^	8.2 ± 0.1 ^cd^
N3	74.7 ± 0.3 ^b^	77.9 ± 0.2 ^c^	82.2 ± 0.3 ^b^	2.8 ± 0.1 ^f^	7.6 ± 0.0 ^d^
N4	76.2 ± 0.1 ^a^	79.4 ± 0.1 ^b^	85.0 ± 0.2 ^a^	1.9 ± 0.1 ^g^	8.7 ± 0.1 ^c^
N5	n.d.	n.d.	n.d.	n.d.	n.d.
N6	n.d.	n.d.	n.d.	n.d.	n.d.

Data represent means ± standard deviations. For each column, values not displaying the same letter are significantly different (*p* < 0.05). To, onset temperature; Tp, peak temperature; Tc, conclusion temperature; ΔH, gelatinization enthalpy. W0–W6: uncooked, and cooked for 5, 10, 15, 20, 25, and 30 min, respectively; N0–N6: Uncooked and cooked for 5, 10, 15, 20, 25, and 30 min, respectively; n.d.; Not detected.

**Table 3:** Pasting properties of uncooked and cooked flours.

Samples	PV (cP)	TV (cP)	BD (cP)	FV (cP)	SB (cP)	PT (min)	PTM (°C)
W0	1300 ± 26 ^j^	848 ± 4 ^j^	452 ± 30 ^i^	1067 ± 13 ^l^	220 ± 16h ^i^	4.0 ± 0.0 ^l^	77.9 ± 0.7 ^h^
W1	2508 ± 28 ^e^	1193 ± 25 ^f^	1315 ± 3 ^e^	1527 ± 16 ^i^	334 ± 10 ^f^	4.0 ± 0.0 ^l^	78.7 ± 0.6 ^g^
W2	3775 ± 15 ^a^	1585 ± 11 ^a^	2190 ± 26 ^a^	1792 ± 24 ^f^	207 ± 35 ^hi^	4.3 ± 0.0 ^k^	81.7 ± 0.0 ^e^
W3	3443 ± 44 ^b^	1520 ± 0 ^c^	1923 ± 44 ^b^	1723 ± 28 ^g^	203 ± 28 ^hi^	4.6 ± 0.0 ^j^	81.5 ± 0.0 ^e^
W4	3073 ± 24 ^c^	1506 ± 6 ^c^	1578 ± 14 ^c^	1694 ± 6 ^h^	191 ± 4 ^j^	4.9 ± 0.0 ^h^	79.1 ± 0.0 ^g^
W5	2522 ± 16 ^e^	1320 ± 9 ^e^	1189 ± 6 ^f^	1551 ± 6 ^i^	231 ± 13 ^h^	4.7 ± 0.0 ^i^	50.1 ± 0.0 ^i^
W6	2618 ± 6 ^d^	1115 ± 13 ^h^	1513 ± 6 ^d^	1392 ± 4 ^j^	286 ± 3 ^g^	1.1 ± 0.0 ^m^	50.3 ± 0.2 ^i^
N0	2175 ± 13 ^f^	1211 ± 9 ^f^	968 ± 9 ^g^	3675 ± 4 ^a^	2473 ± 6 ^a^	5.1 ± 0.0 ^f^	79.9 ± 0.0 ^f^
N1	1842 ± 15 ^h^	1340 ± 11 ^e^	502 ± 4 ^h^	2568 ± 5 ^c^	1237 ± 6 ^b^	5.1 ± 0.0 ^g^	79.2 ± 0.1 ^g^
N2	2096 ± 9 ^g^	1558 ± 6 ^b^	538 ± 4 ^h^	2813 ± 8 ^b^	1260 ± 9 ^b^	5.3 ± 0.0 ^e^	81.6 ± 0.0 ^e^
N3	1749 ± 9 ^i^	1415 ± 11 ^d^	348 ± 18 ^j^	2454 ± 5 ^d^	1047 ± 6 ^c^	5.6 ± 0.0 ^d^	83.2 ± 0.0 ^d^
N4	1306 ± 8 ^j^	1158 ± 4 ^g^	147 ± 3 ^k^	1914 ± 5 ^e^	758 ± 4 ^d^	5.9 ± 0.0 ^c^	84.7 ± 0.1 ^c^
N5	912 ± 8 ^k^	887 ± 6 ^i^	24 ± 1 ^l^	1316 ± 4 ^k^	438 ± 1 ^e^	6.6 ± 0.0 ^b^	88.0 ± 0.0 ^b^
N6	618 ± 4 ^l^	579 ± 5 ^k^	47 ± 1 ^l^	938 ± 9 ^m^	359 ± 4 ^f^	7.0 ± 0.0 ^a^	92.2 ± 0.3 ^a^

Data represent means ± standard deviations. For each column, values not displaying the same letter are significantly different (*p* < 0.05). PV, peak viscosity; TV, trough viscosity; BD, breakdown viscosity; FV, final viscosity; SB, setback viscosity; PTM, Pasting temperature; PT, peak time. W0–W6: uncooked, and cooked for 5, 10, 15, 20, 25, and 30 min, respectively; N0–N6: Uncooked and cooked for 5, 10, 15, 20, 25, and 30 min, respectively.
